# A Multimodal Multi-Objective Feature Selection Method for Intelligent Rating Models of Unmanned Highway Toll Stations

**DOI:** 10.3390/biomimetics9100613

**Published:** 2024-10-10

**Authors:** Zhaohui Gao, Huan Mo, Zicheng Yan, Qinqin Fan

**Affiliations:** 1 Intelligent Transportation System Research Center, Southeast University, Nanjing 211189, China; 101005502@seu.edu.cn; 2 Logistics Research Center, Shanghai Maritime University, Shanghai 201306, China; 202330510271@stu.shmtu.edu.cn (H.M.); 202230510129@stu.shmtu.edu.cn (Z.Y.)

**Keywords:** intelligent transportation, evolutionary computation, feature selection, multimodal multi-objective optimization

## Abstract

To facilitate the intelligent classification of unmanned highway toll stations, selecting effective and useful features is pivotal. This process involves achieving a tradeoff between the number of features and the classification accuracy while also reducing the acquisition costs of features. To address these challenges, a multimodal multi-objective feature selection (MMOFS) method is proposed in the current study. In the MMOFS, we utilize a multimodal multi-objective evolutionary algorithm to choose features for the unmanned highway toll station classification model and use the random forest method for classification. The primary contribution of the current study is to propose a feature selection method specifically designed for the classification model of unmanned highway toll stations. Experimental results using actual data from highway toll stations demonstrate that the proposed MMOFS outperforms the other two competitors in terms of PSP, HV, and IGD. Furthermore, the proposed algorithm can provide decision-makers with multiple equivalent feature selection schemes. This approach achieves a harmonious balance between the model complexity and the classification accuracy based on actual scenarios, thereby providing guidance for the construction of unmanned highway toll stations.

## 1. Introduction

Highways are important components of the transportation system. However, congestion on expressways can lead to significant environmental and socio-economic issues [1]. Analyzing highway congestion reveals that severe congestion is often caused by the presence of manual toll booths [2]. Numerous studies have been studying the different aspects of highway tolls. For example, Velarde et al. [3] set motorway toll prices to control traffic flow and reduce congestion. Moreover, Gui et al. [4] adjusted the capacity of toll booths to control traffic flow. Ling et al. [5] proposed an optimization model for toll station lane configuration to minimize the total cost, which contains toll station operating cost and delay cost, combined with the queuing theory. The results demonstrate that the proposed method is effective in reducing congestion at toll booths. Rota et al. [6] highlighted that the intelligent traffic system (ITS) combined with emerging unmanned ground vehicles (UGVs) and novel tolling methods can significantly alleviate these bottlenecks. Souza [7] pointed out that the free-flow system, an electronic system for automatic vehicle identification, improved toll collection efficiency by electronically identifying and charging passing vehicles automatically. The results indicate that this system can improve toll collection efficiency. Therefore, implementing free-flow tolling is an effective approach to reduce highway congestion and improve traffic efficiency. However, due to various special situations at highway toll stations, such as vehicles benefiting from the “green channel” preferential policy and incorrect vehicle information, it is not realistic to have all highway toll stations fully unmanned. This underscores the need for intelligent rating models using various machine learning methods to assess the unmanned level of highway toll stations and determine the types of each unmanned highway toll station. Additionally, in the context of future developments in intelligent transportation methods and technologies, the intelligent classification model of unmanned highway toll stations can be valuable for prioritizing the implementation of free-flow tolling. 

To improve the classification performance of machine learning methods in the field of intelligent transportation, feature selection is a fundamental step, as this process effectively reduces data dimensionality, shortens learning time, and improves classification performance [8]. The feature selection methods have been widely used in the field of intelligent transportation. For example, Kandiri et al. [9] used an interconnected optimization algorithm for feature selection to identify the best set of features, thereby improving the prediction accuracy of travel time in intelligent transportation systems. Zheng et al. [10] proposed a hybrid intelligent algorithm-based feature selector to optimize original state vectors. The results verify that this feature selection method can significantly enhance the predicted accuracy of the model. Liu et al. [11] proposed a community-based dandelion algorithm (CDA) to solve the traffic flow prediction problem. The experiment results show that the proposed method can select suitable features via the coding and decoding strategies and improve the prediction accuracy by 5–16%. For a large training set, Pareek et al. [12] proposed a feature selection method using a hybrid slime procedure and whale optimization, which is used for the selection of important variables and the elimination of duplicates, and then the extreme machine learning (ELM) is used for prediction. The experimental results demonstrated that the proposed method has good prediction performance and high prediction accuracy. Therefore, the feature selection can improve classification accuracy. Similar to the above studies, Wahab et al. [13] introduced a feature selection algorithm to delete irrelevant and abnormal features in advance and to cluster the intelligent features of the vehicle’s self-organizing network. Almutlaq et al. [14] proposed a hybrid feature selection method that combines three filter-based methods and two wrapper-based methods. The proposed method aids in eliminating irrelevant features in the intrusion detection of intelligent transportation systems. The results show that the method has faster prediction speed and improved classification accuracy. Kavitha et al. [15] proposed an optimized YOLOv2 model for vehicle detection and classification, incorporating a multi-layer feature fusion strategy to improve the effectiveness of feature selection. The experimental results demonstrate that the proposed method can achieve accurate vehicle classification. In addition to using feature selection methods to improve the classification performance of models, many studies also investigate classification models in the field of transportation [16]. For instance, Arinaldi et al. [17] proposed a classification method based on support vector machines (SVMs) and faster region convolutional neural networks (RCNNs) to identify vehicles. Sarikan et al. [18] used a classification method based on K-nearest neighbors (KNNs) and decision trees to classify vehicles. Like the above studies, Barreyro et al. [19] utilized an AlexNet convolutional neural network to identify automatic vehicles. The results show that the classification accuracy of the proposed algorithm can meet actual requirements. Machine learning classifiers have been applied for vehicle classification. Trivedi et al. [20] pointed out that the application of machine learning methods for vehicle classification is effective.

From the above-mentioned studies, feature selection is often treated as a single-objective optimization problem. However, it can also be viewed as a multi-objective combinatorial optimization problem, mainly involving two objectives: (1) the number of selected features and (2) the classification accuracy. To solve this problem, Liang et al. [21] proposed a feature selection algorithm based on a multi-objective evolutionary algorithm (FS-MOEA), in which the information gain (IG)–analytic hierarchy process (AHP) is used to prioritize the search for the best feature subset. The experimental results verify that the proposed algorithm achieves higher classification accuracy and lower computational complexity, making it suitable for use in intrusion detection systems (IDSs) in vehicular self-organizing networks (VANETs). Bohrer et al. [22] proposed a hybrid feature selection approach using a multi-objective genetic algorithm. The proposed method uses a multi-objective genetic algorithm to generate a feature set by combining and optimizing the feature sets generated by other conventional feature selection methods. The experimental results demonstrate that the proposed algorithm is highly effective. To further improve the algorithm performance in solving multi-objective feature selection problems, Zhang et al. [23] proposed a novel information gain-based evolutionary algorithm for multi-objective feature selection. Note that the proposed algorithm employs the information gain as a metric to evaluate the contribution of features. Dong et al. [24] proposed a many-objective optimization-based multi-label feature selection algorithm (MMFS) in which the real number encoding method is used to design new crossover operators and mutation operators. The results demonstrate the superiority of the algorithm. Xue et al. [25] proposed a multi-objective binary genetic algorithm integrating an adaptive operator selection mechanism (MOBGA-AOS) for feature selection. To fully utilize the search capabilities of different crossover operators in the genetic algorithms (GAs), the proposed algorithm uses five different crossover operators to generate a set of optimal feature subsets characterized by small size and high classification accuracy. The experimental results show that the proposed algorithm has significant advantages in handling large-scale datasets.

Besides conflicting objectives, real-world feature selection also exhibits multimodality [26,27]. In other words, the feature selection can be considered as a multimodal multi-objective optimization problem. Compared with ordinary multi-objective optimization problems, multimodal multi-objective optimization not only requires finding a high-quality Pareto front approximation in the objective space but also demands finding sufficient equivalent Pareto solutions in the decision space [28]. To solve the above issue, Yue et al. [29] proposed a multimodal multi-objective algorithm for feature selection. The experiments verify that the proposed algorithm can provide a high-quality feature subset without significantly reducing the classification accuracy. Hu et al. proposed a novel multimodal niching particle swarm optimization (MNPSO) algorithm to select features. The proposed algorithm employs the crowding distance and species clustering methods to divide the population to improve population diversity. The experimental results demonstrate that the proposed algorithm can effectively find more multimodal feature selection solutions [30]. Subsequently, Liang et al. [31] proposed a multimodal multi-objective genetic algorithm for feature selection, which can successfully find equivalent feature subsets on different datasets. Wang et al. [32] proposed a multi-objective differential evolution approach in which a novel feature relevance-based population initialization method is proposed to improve the search performance. The experimental results show that the proposed algorithm can obtain a greater number of higher-quality feature subsets. Jha et al. [33] used a multimodal multi-objective optimization algorithm based on a ring topology structure to perform filtering feature selection based on factors such as mutual information and redundancy between features, ultimately selecting feature subsets with minimum redundancy and maximum correlation. The experimental results show that the proposed algorithm can not only provide a larger number of equivalent feature subsets but also has better or similar prediction accuracy when compared with other feature selection methods.

From existing studies, although multimodal multi-objective feature selection methods have been used in various fields, they have rarely been utilized for feature selection problems in intelligent transportation systems, especially for highway toll stations. To find more equivalent feature selection schemes for the unmanned highway toll rating model and investigate the relationship between the model classification accuracy and the number of features, a multimodal multi-objective feature selection (MMOFS) method is introduced in the current study. In the MMOFS, a competitive multimodal multi-objective evolutionary algorithm is employed to explore diverse feature selection schemes in the objective space, elucidating the relationship between the model classification accuracy and the number of selected features. Moreover, it is employed to discover more equivalent solutions in the decision space, suitable for adapting to different scenarios and requirements. Additionally, the random forest (RF) algorithm [34] is used to evaluate the effectiveness of feature selection solutions. The performance of the MMOFS is compared with that of two selected algorithms on a real-world dataset. The results demonstrate that the proposed algorithm can provide decision-makers with high-quality and diverse feature selection schemes.

The main contributions of the present study are as follows:

(1) To reduce highway congestion and achieve free-flow tolling, this study uses artificial intelligence methods to develop an intelligent rating model for unmanned highway toll stations based on real-world toll station data. Moreover, the level of unmanned highway toll stations is categorized into three levels.
(2) A multimodal multi-objective feature selection method is employed to facilitate feature selection, providing multiple high-quality and equivalent feature selection schemes. This approach aids decision-makers in model development under various conditions while reducing modeling costs, thereby providing a reliable basis for the construction of unmanned toll stations.

## 2. Methodology

In multi-objective feature selection problems, there is often a conflict between the number of selected features (*f***_1_**) and the classification accuracy of the model (*f***_2_**). Therefore, they can be considered as two conflicting optimization objectives, which can be defined as follows:

(1) $$\left\{\begin{array}{l} \min f_{1}\left(x\right)=\frac{1}{D}\sum_{i=1}^{D}\left|x_{i}\right|\\ \min f_{2}\left(x\right)=ER\left(x\right) \end{array}\right.,$$

where *x* represents a feature subset; *D* denotes the total number of original features; ER is the classification error rate. For the multi-objective feature selection, different feature subsets may be equivalent in some scenarios. This means that selecting different feature schemes may not affect the classification performance of models. Note that this equivalence can significantly reduce the cost of acquiring features. Therefore, the feature selection problems are treated as multimodal multi-objective problems to effectively select features for the intelligent rating model of highway toll stations. Additionally, compared with the filter and embedded methods, the wrapper method can select superior feature subsets [35]. Therefore, the wrapper method is utilized to select features. 

### 2.1. Encoding and Decoding Methods

**Figure 1:**
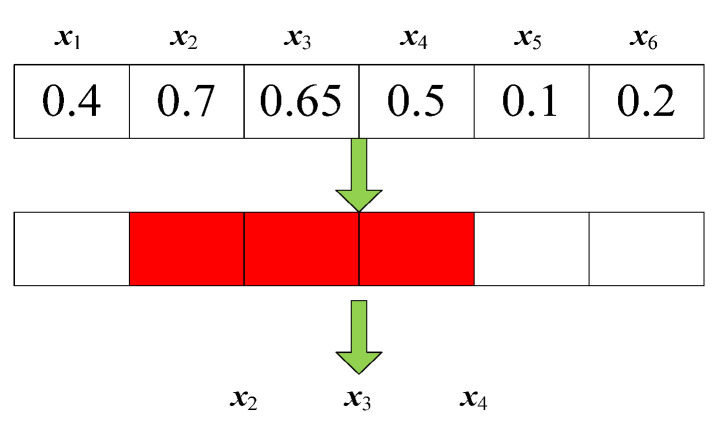
The encoding and decoding method (red color indicates the selected feature).

Because the particle swarm optimization, used as the search engine, is suitable for solving continuous optimization problems, it cannot be used to select features in the present study. To solve this issue, like the individual encoding method in Refs. [36,37], the current study also uses the real number encoding method. Namely, each decision variable ranges between 0 and 1. If a decision variable is greater than or equal to 0.5, the corresponding feature is selected; otherwise, this feature will be removed. The specific encoding and decoding process for individuals is illustrated in Figure 1. As shown in Figure 1, this individual has six features. Among them, features *x*_2_, *x*_3_, and *x*_4_ are greater than or equal to 0.5, indicating that these three features will be selected. Conversely, features *x*_1_, *x*_5_, and *x*_6_ are less than 0.5 and are thus not selected. 

### 2.2. IDMMPSO

Since the improved discrete multimodal multi-objective particle swarm optimization (IDMMPSO) algorithm [36] is both effective for solving discrete multimodal multi-objective optimization problems and easily accessible, it is used to address the multimodal multi-objective feature selection problems. The main steps of the IDMMPSO are as follows (Algorithm 1): Line 1 is to generate an initial population. Subsequently, compute the fitness function values of all initial individuals (line 2). The INSCD method [36], which uses the Hamming distance to compute the crowding distance in the non-dominated_scd_sort method, is employed to sort all individuals into two archives, i.e., ***HOA*** and ***NOA*** (line 5). Select the first individuals from ***HOA***{*i*}, denoted as *pbest_i_*. Moreover, choose the first individual from ***NOA***{*i*} and denote it as *nbest_i_*. The velocity formula is used to update the *i*-th particle (see line 7). Lines 8–14 are to convert real-number into 0–1 integer. After generating *i*-th individuals, calculate its fitness function value and save it to ***HOA***{*i*}. Line 16 is to use the INSCD method to update the ***HOA***{*i*}. Subsequently, the environmental selection method is utilized to choose non-dominated individuals from ***HOA***{*i* − 1}, ***HOA***{*i*}, and ***HOA***{*i* + 1}, and save them to ***NOA***{*i*}. Steps in lines 4–19 are repeated until *G* = *G*_max_. Finally, output all non-dominated individuals in the ***NOA***.
**Algorithm 1** IDMMPSO**Input:** the population size, *NP*; the maximum number of generations, *G*_max_; the dimension of individual, *D*; the historical optimal archive, ***HOA***; the neighbor optimal archive, ***NOA***. 1:          Generate an initial population ***P***^0^; set *G* = 1; 2:          Compute the fitness function values of all individuals in ***P***^0^; 3:          **while** *G* < *G*_max_ **do** 4:                     **for** *i* = 1:* NP* **do** 5:                          The INSCD method is used to sort all individuals in both ***HOA*** and                              ***NOA***; 6:                          Select the first individuals from *HOA*{*i*} and* NOA*{*i*}, respectively, and                              denote them as *pbest_i_* and *nbest_i_*; 7:                          the velocity of the *i*-th particle is updated via                              $v_{i}^{G+1}=\omega \cdot v_{i}^{G}+c_{1}r_{1}(pbest_{i}^{G}-x_{i}^{G})+c_{2}r_{2}(nbest_{i}^{G}-x_{i}^{G})$; 8:                          **for** *j* = 1: *D* **do** 9:                                **if** $rand\;<\;\mathrm{logsig}(v_{i,j}^{G+1})$, **then** 10:                                  $x_{i,j}^{G+1}=1$; 11:                             **else** 12:                                  $x_{i,j}^{G+1}=0$; 13:                             **end** **if** 14:                        **end** **for** 15:                        Calculate the fitness function value of the $x_{i}^{G+1}$ and save it to ***HOA***{*i*}; 16:                        The INSCD method is utilized to update the ***HOA***{*i*}; 17:                        The environmental selection method is used to choose non-dominated                              individuals from ***HOA***{*i* − 1}, ***HOA***{*i*}, and ***HOA***{*i* + 1} and save them to                              ***NOA***{*i*}; 18:                  **end** **for** 19:                      *G* = *G* + 1; 20:        **end** **while** **Output:**  All non-dominated individuals in ***NOA***.

### 2.3. Random Forest

The RF proposed by Breiman [38] is a competitive and effective classifier method. The pseudocode of the RF is shown in Algorithm 2. The first step is to generate *M* randomized training sets (see line 1). For the *k*-th dataset (denoted as ***D****_k_*), randomly select a subset of features (i.e., ***F***′) from original features. Note that $\left|F^{\prime }\right|$ should be less than *N*. Subsequently, line 4 is to build a decision tree using ***D****_k_* and ***F***′. After building all the decision trees, a voting mechanism is employed to aggregate all their predicted results.
**Algorithm 2** RF**Input**: the dataset, **D**; the number of trees, *M*; the number of features in the original dataset, *N*; 1:**for** *k* = 1: *M* **do**2:      The bootstrap sampling method is used to generate a training set, ***D**_k_*;3:        Randomly select a subset of features, ***F***′; $\left|F^{\prime }\right|$ < *N* 4:        Build a decision tree (***T**_k_*) based on the ***D***_k_ and ***F***′;5:  **end for**6:  Aggregate predicted results from all decision trees;7:**Output**: the final classification predicted result. 

### 2.4. Overall Implementation of the Proposed Algorithm

In this study, a multimodal multi-objective feature selection method (MMOFS) is proposed to effectively develop an intelligent classification model for unmanned highway toll stations and to obtain multiple equivalent feature subsets that accommodate diverse scenario demands and varying feature acquisition costs. In the proposed MMOFS, its main steps are the same as the IDMMPSO, with the primary differentiation found in the computation of objective functions. Specifically, one of the objective functions in the MMOFS is the classification accuracy obtained from the RF algorithm. The main steps of the MMOFS are shown in Algorithm 3 in Section 2.2. Line 1 aims to produce an initial population. The objective function values (i.e., classification accuracy) of all individuals in the ***P***^0^ are obtained by the RF algorithm. Note that the other objective function is the number of selected features. Lines 3–19 describe the use of IDMMPSO to identify feature selection schemes for the highway toll station rating model, as detailed in Section 2.2. Finally, output all feature selection schemes.
**Algorithm 3** MMOFS**Input:** the population size, *NP*; the maximum number of generations, *G*_max_; the dimension of individual, *D*; the historical optimal archive, ***HOA***; the neighbor optimal archive, ***NOA***. 1:          Generate an initial population ***P***^0^; set *G* = 1; 2:          Compute the fitness function values of all individuals in ***P***^0^ via the RF;3:          ***while*** *G* < *G*_max_ ***do*** 4:          **for** *i* = 1: *NP* **do** 5:                The INSCD method is utilized to rank all individuals in both ***HOA*** and ***NOA***; 6:                Choose the first individual from ***HOA***{*i*} and ***NOA***{*i*}, respectively, and denote them as *pbest_i_* and *nbest_i_*; 7:                $v_{i}^{G+1}=\omega \cdot v_{i}^{G}+c_{1}r_{1}(pbest_{i}^{G}-x_{i}^{G})+c_{2}r_{2}(nbest_{i}^{G}-x_{i}^{G})$ is used to update the velocity of                   the *i*-th individual; 8:             **for** *j* = 1: *D* **do** 9:                     **if** $rand\;<\;\mathrm{logsig}(v_{i,j}^{G+1})$, **then** 10:                            $x_{i,j}^{G+1}=1$; 11:                     **else** 12:                            $x_{i,j}^{G+1}=0$; 13:                     **end if** 14:             **end for** 15:                Calculate the fitness function value of the $x_{i}^{G+1}$ using the RF algorithm and save it to ***HOA***{*i*}; 16:                All individuals in the ***HOA***{*i*} are ranked using the INSCD method. Moreover, a                      certain number of individuals are selected and saved to ***HOA***{*i*}; 17:                Select non-dominated individuals from ***HOA***{*i* − 1}*,* ***HOA***{*i*}, and ***HOA***{*i* + 1}                     using the environmental selection method and store them in ***NOA***{*i*}; 18:             **end for** 19:          **end while** **Output:** All the non-dominated individuals in ***NOA***. 

## 3. Experimental Results and Analyses

### 3.1. Datasets

Real highway toll station data are used in the present study, which includes data from 13 highway toll stations, comprising 117 data samples and 23 features. Note that we only use 12 features listed in Table 1 due to the lack of relevant data for 11 out of the 23 features. Moreover, the unmanned highway toll stations are classified into three levels: (1) unmanned exit or entrance (U1); (2) unmanned exit and entrance (U2); (3) unmanned highway toll station (U3). For U1, the highway toll station has either the exit or entrance unmanned, with remote monitoring employed for the unattended exit or entrance. U2 involves an unmanned exit and entrance, with staff conducting remote monitoring from a smart cloud storage facility either in the station building or nearby. In contrast, U3 toll stations do not have a monitoring center. 

### 3.2. Parameter Settings

The actual dataset is randomly split into training and test sets, with the training set comprising 80% of the total samples and the test set comprising the remaining 20%. Moreover, the three-fold cross-validation is used to train the model. For the RF algorithm, the number of trees and the minimum number of samples required for a leaf node are set to twenty and two, respectively. Additionally, the parameter settings in the IDMMPSO algorithm are the same as those in Ref. [36].

### 3.3. Comparisons with Competitive Algorithms

To demonstrate the feature selection performance of the proposed algorithm, it is compared with two other algorithms: MOPSO-based [39] and NSGA-II-based [40] random forest algorithms, named MOPSO-RF and NSGA-II-RF. Moreover, the performances of these compared algorithms are evaluated using three commonly used multi-objective performance metrics: the Pareto set proximity(PSP) [41], the hypervolume (HV) [42], and inverted generational distance (IGD) [43]. Note that the IGD value is computed using the Pareto front (PF) approximation obtained from each compared algorithm and a reference PF, which is selected from all the non-dominated solutions of all the compared algorithms via the environmental selection method [36]. Additionally, each compared algorithm is run five times on this feature selection problem. According to Ref. [44], the unpaired *t*-test method is used to analyze the performance of all the compared algorithms since the sample is unpaired and meets the normal distribution. The symbols “+”, “−”, and “≈” represent that the proposed MOPSO-RF is superior to, inferior to, and comparable with its competitors, respectively.

The mean and standard deviation values for all compared algorithms are shown in Table 2. It can be observed from Table 2 that the performance of the MOPSO-RF is significantly better than that of the other two competitors in terms of the PSP. Because the PSP is mainly used to assess the performance of the compared algorithm in the decision space, we can conclude that the proposed algorithm can find more equivalent feature selection solutions when compared with the MOPSO-RF and the NSGA-II-RF. Moreover, Table 2 indicates that the MOPSO-RF outperforms two compared algorithms in a statistical method in terms of HV and IGD. This means that the proposed algorithm is able to achieve higher-quality feature selection schemes. Therefore, it can be concluded that the proposed algorithm is an effective and competitive approach to selecting features for decision-makers. 

**Figure 2:**
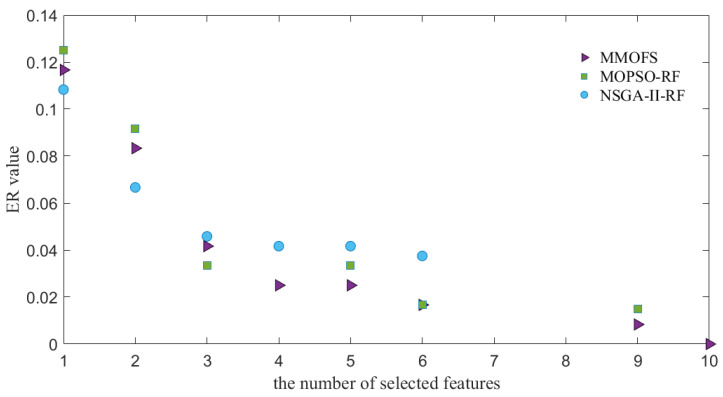
PF approximations of three compared algorithms.

**Figure 3:**
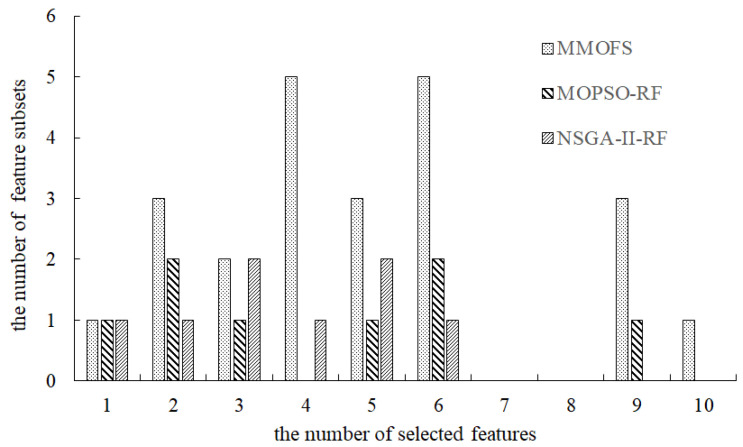
The number of feature subsets obtained by three compared algorithms under different cases.

To visually demonstrate the performance of the MMOFS, the PFs and Pareto sets (PSs) obtained by the three compared algorithms are shown in Figure 2 and Figure 3. From Figure 2, it is evident that, except for cases where the number of selected features is one, two, or three, the proposed algorithm can achieve a high-quality PF approximation when compared with the NSGA-II-RF. Moreover, the proposed algorithm consistently identifies high-quality solution sets in all cases when compared with the MOPSO-RF. Finally, Figure 2 also reveals that, for the intelligent rating model of unmanned highway toll stations, there is an inherent trade-off between the model accuracy and the number of selected features. From Figure 3, we can observe that, compared with the MOPSO-RF and the NSGA-II-RF, the MMOFS can find more equivalent feature subsets in most cases when the number of selected features is the same. The main reason may be that the INSCD method can assist the proposed algorithm in preserving the population diversity and locating more equivalent solutions. This indicates that the proposed algorithm has stronger feature selection capability and provides decision-makers with more equivalent feature selection schemes for use in different scenarios. Therefore, it can be concluded that the proposed algorithm is an effective approach to select features. 

### 3.4. Multimodal Analysis of Feature Selection Schemes

To further illustrate the effectiveness of the proposed algorithm, this experiment analyzes the multimodality of feature selection schemes. All feature selection schemes obtained by the proposed algorithm are presented in Table 3. We can observe from Table 3 that for the same number of selected features and model classification accuracy, the proposed algorithm can provide equivalent feature selection schemes in most cases. For example, feature selection schemes {*x*_1_, *x*_4_, *x*_7_, *x*_9_, *x*_11_} and {*x*_4_, *x*_5_, *x*_7_, *x*_11_, *x*_12_} are equivalent when the number of features is five, and the ER value is 0.02. However, given that the acquisition cost of feature *x*_12_ (i.e., peak daily volume) is higher than that of other features in practice, we can adopt feature selection solution {*x*_1_, *x*_4_, *x*_7_, *x*_9_, *x*_11_} to build the intelligent rating model of highway toll stations without increasing its complexity and compromising its classification performance. This significantly reduces the cost of feature acquisition. Additionally, not all highway toll stations have complete special condition data; thus, equivalent feature selection schemes can be used to evaluate their levels. It can be concluded that the proposed algorithm can find equivalent feature selection schemes, thereby reducing modeling costs.

### 3.5. Influence of the Selected Performance Indicators 

In the original MMOFS, the ER is used to assess the effectiveness of features. To analyze the influence of performance metrics, the Kappa coefficient method and macro-averaged precision, which are used to evaluate the classification performance of algorithms on multi-class problems, are employed in the MMOFS, named as the MMOFS-1 and MMOFS-2. Moreover, the parameter settings of the MMOFS-1 and the MMOFS-2 are the same as in Section 3.2.

**Figure 4:**
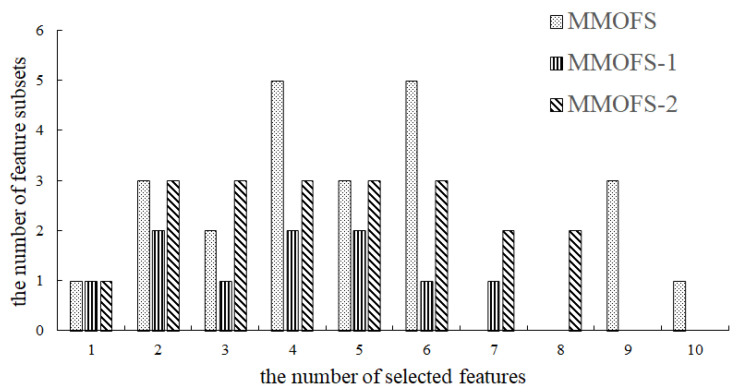
The number of feature subsets obtained by the MMOFS using different performance indicators.

The results of the MMOFS and its two variants are illustrated in Figure 4. We can observe from Figure 4 that using different performance indicators in the proposed algorithm can lead to different feature selection schemes. Therefore, the performance of the proposed algorithm is influenced by the selected performance metric. In other words, like other feature selection methods, the proposed MMOFS is also sensitive to specific models. Additionally, Figure 4 indicates that the ER can help the proposed algorithm find more feature selection solutions; thus, the ER is used in the proposed algorithm. 

## 4. Discussion

The unmanned highway toll station can greatly improve traffic efficiency, but its construction must determine the unmanned level based on actual conditions. Therefore, developing a classification model for unmanned highway toll stations is essential, as its performance can be substantially affected by feature selection schemes. In this study, a multimodal multi-objective feature selection (MMOFS) approach is introduced to provide a set of feature selection schemes for decision-makers. 

The findings in Section 3.3 demonstrate that the MMOFS is superior to the NSGA-II and the MOPSO in finding feature selection solutions. The results can be discussed from two perspectives: (1) For the decision space, the PSP values in Table 2 show that the MMOFS can assist the RF in finding more equivalent feature schemes when compared with the other two competitors. This can not only improve the algorithm’s adaptability to different scenarios, such as missing data, but also reduce the cost of feature acquisition, including the use of expensive data collection equipment. (2) In terms of the objective space, we can observe from Table 2 that the proposed MMOFS can help the RF achieve better feature selection solutions, which can influence the classification accuracy and the model complexity. Therefore, the MMOFS is an effective method for selecting features for the classification model of unmanned highway toll stations. Additionally, the application of the MMOFS is not limited to highway toll stations; it is also suitable for other classification challenges in the transportation field and beyond. 

The results in Section 3.4 show that the proposed algorithm not only achieves high classification accuracy but also finds more equivalent feature schemes. Clearly, the RF method, incorporated into the MMOFS, exhibits high classification accuracy, providing a solid foundation for constructing unmanned highway toll stations. This allows decision-makers to easily determine the unmanned level of highway toll stations via actual conditions. Moreover, the proposed MMOFS can locate more equivalent feature selection schemes, which can improve the adaptability and applicability of classifiers, as well as their decision-making efficiency. Additionally, for different highway toll stations facing issues of insufficient historical data, we can adopt equivalent feature selection schemes based on existing data for modeling. This can significantly reduce the investment costs of additional equipment and shorten the modeling time. Finally, we can observe that the number of selected features conflicts with the classification performance; specifically, the classification performance of the algorithm improves as the number of selected features increases. Considering that each highway toll station has different data collection devices, each station can select a feature scheme specifically tailored to its unique conditions, allowing for a customized approach to constructing the classification model. 

The MMOFS is an effective and competitive feature selection method. However, it has some limitations: (1) Although the proposed MMOFS can identify a larger quantity and higher quality of feature selection schemes when compared with the other two competitors, it fails to find any solutions when the number of selected features is seven or eight. Therefore, the search performance of the proposed algorithm needs further improvement. (2) Data imbalance presents a significant challenge for classification problems, and the proposed algorithm does not currently address this issue, which may affect its overall applicability in such scenarios. (3) Different fields require distinct feature selection methods. However, this study focuses solely on a particular scenario, limiting the capability of the MMOFS to evolve autonomously. 

## 5. Conclusions

To find more equivalent and diverse feature selection schemes for unmanned highway toll station rating models that can adapt to changing scenarios and reduce feature acquisition costs, the multimodal multi-objective feature selection (MMOFS) method is proposed in the current study. In the MMOFS, an improved multimodal multi-objective evolutionary algorithm is utilized to find feature selection schemes. Moreover, the RF algorithm is used to evaluate the effectiveness of these feature selection schemes. Based on real-world highway toll station data, the proposed algorithm is compared with two other algorithms. The experimental results demonstrate that the proposed algorithm can identify more high-quality feature selection schemes due to its effective maintenance of population diversity. This provides decision-makers with diverse feature selection options and can reduce the cost of feature acquisition, thereby offering valuable references for the construction of unmanned highway toll stations.

## Figures and Tables

**Table 1:** 12 Selected features.

	Definition	Type
*x*_1_	ETC card mismatch	Discrete
*x*_2_	U-type special condition	Discrete
*x*_3_	Vehicle type mismatch	Discrete
*x*_4_	Overlimit	Discrete
*x*_5_	Cash transaction count	Discrete
*x*_6_	ETC malfunction	Discrete
*x*_7_	Green priority exception	Discrete
*x*_8_	Manual gate barrier	Discrete
*x*_9_	No ETC	Discrete
*x*_10_	Weighing fault	Discrete
*x*_11_	Axle load modification	Discrete
*x*_12_	Peak daily volume	Discrete

**Table 2:** Mean and standard deviation values of three feature selection methods in terms of PSP, HV, and IGD.

	NSGA-II-RF Mean(std)		MOPSO-RF Mean(std)		MMOFS Mean(std)
PSP	8.49 × 10^1^ (3.94 × 10^1^)	+	7.04 × 10^1^ (9.76 × 10^0^)	+	1.89 × 10^2^ (3.99 × 10^1^)
HV	1.15 × 10^2^ (3.67 × 10^0^)	+	1.19 × 10^2^ (4.37 × 10^0^)	+	1.29 × 10^2^ (2.04 × 10^0^)
IGD	1.31 × 10^0^ (2.96 × 10^−1^)	+	7.64 × 10^−1^ (3.06 × 10^−1^)	+	7.37 × 10^−1^ (2.54 × 10^−1^)
+		3		3	
−		0		0	
≈		0		0	

**Table 3:** Feature subsets obtained by the proposed algorithm.

The Number of Selected Features	ER Value	Feature Subset
1	0.12	*x*_7_
2	0.08	*x*_7_, *x*_11_
		*x*_7_, *x*_9_
		*x*_9_, *x*_12_
3	0.04	*x*_2_, *x*_7_, *x*_11_
		*x*_7_, *x*_9_, *x*_11_
4	0.03	*x*_4_, *x*_6_, *x*_7_, *x*_12_
		*x*_1_, *x*_7_, *x*_9_, *x*_11_
		*x*_1_, *x*_7_, *x*_10_, *x*_11_
		*x*_1_, *x*_3_, *x*_7_, *x*_11_
		*x*_4_, *x*_5_, *x*_7_, *x*_11_
5	0.02	*x*_1_, *x*_4_, *x*_7_, *x*_9_, *x*_11_
		*x*_4_, *x*_5_, *x*_7_, *x*_11_, *x*_12_
		*x*_2_, *x*_4_, *x*_7_, *x*_8_, *x*_11_
6	0.016	*x*_1_, *x*_5_, *x*_7_, *x*_10_, *x*_11_, *x*_12_
		*x*_1_, *x*_3_, *x*_4_, *x*_7_, *x*_9_, *x*_11_
		*x*_2_, *x*_3_, *x*_4_, *x*_7_, *x*_8_, *x*_11_
		*x*_1_, *x*_4_, *x*_7_, *x*_9_, *x*_10_, *x*_11_
		*x*_1_, *x*_2_, *x*_3_, *x*_7_, *x*_9_, *x*_11_
9	0.008	*x*_1_, *x*_3_, *x*_4_, *x*_5_, *x*_6_, *x*_7_, *x*_10_, *x*_11_, *x*_12_
		*x*_1_, *x*_2_, *x*_4_, *x*_5_, *x*_7_, *x*_8_, *x*_9_, *x*_10_, *x*_11_
		*x*_1_, *x*_4_, *x*_5_, *x*_6_, *x*_7_, *x*_8_, *x*_10_, *x*_11_, *x*_12_
10	0	*x*_2_, *x*_3_, *x*_4_, *x*_5_, *x*_6_, *x*_7_, *x*_9_, *x*_10_, *x*_11_, *x*_12_

## Data Availability

Data supporting reported results are available from the authors upon reasonable request.
